# Fabrication and *in vitro* cytocompatibility evaluation of porous bone scaffold based on cuttlefish bone-derived nano-carbonated hydroxyapatite reinforced with polyethylene oxide/chitosan fibrous structure

**DOI:** 10.1039/d4ra08457h

**Published:** 2025-02-17

**Authors:** Musyafa Riziq Habiburrohman, Muhammad Amir Jamilludin, Nilam Cahyati, Nendar Herdianto, Yusril Yusuf

**Affiliations:** a Department of Physics, Faculty of Mathematics and Natural Sciences, Universitas Gadjah Mada Yogyakarta 55281 Indonesia yusril@ugm.ac.id; b Research Centre for Advanced Material, National Research and Innovation Agency (BRIN) South Tangerang 15314 Indonesia

## Abstract

A novel porous bone scaffold based on nano-carbonated hydroxyapatite reinforced with fibrous-like structured polyethylene oxide/chitosan network (nCHA/PEO/CS) was introduced and fabricated *via* freeze-drying. Prior to this, the nCHA was synthesized through a hydrothermal reaction based on cuttlefish bone (CFB, *Sepia officinalis*). The raw cuttlefish bone (raw-CFB) was first decomposed to obtain cuttlefish bone-derived calcium oxide (CaO-CFB) by calcination at 1000 °C, which was used for synthesizing nCHA. The chemical composition analysis showed that the nCHA formed AB-type CHA with a high carbonate content of 7.38 wt%, which is in the range of carbonate content in native bone (2–9 wt%). The Ca/P molar ratio of nCHA was 1.712, very close to the Ca/P of biological apatite of 1.71. Morphological analysis revealed that nCHA consists of nanosized particles, potentially offering a large surface area to volume to promote ion exchange and cell interaction. The excellent physicochemical and morphological properties of nCHA proposed suitability as a bone scaffold precursor combined with PEO and CS. The nCHA/PEO/CS scaffolds were freeze-dried with varying PEO/CS concentrations. Physicochemical analysis indicated that increasing the PEO/CS concentration decreased the crystallinity of the scaffold, causing it to be lower than the nCHA crystallinity, which may be beneficial for cell growth. Morphological analysis revealed that the scaffold structure comprised nCHA cross-linked within a fibrous-like structured PEO/CS network, which appropriately mimics the fibrous structure of extracellular matrix (ECM) in natural bone. However, the nCHA/PEO/CS-11 scaffold formed more appropriate pores with suitable porosity for cell development, blood vessel formation, and nutrient perfusion. The nCHA/PEO/CS-11 scaffold also demonstrated sufficient compressive strength and good swelling behavior, which may favor bone regeneration. The nCHA/PEO/CS-11 scaffold demonstrated high cytocompatibility and facilitated the adherence of MC3T3E1 cells on the scaffold surface. The nCHA/PEO/CS-11 scaffold also promoted cell osteogenic differentiation. Owing to its desirable and suitable characteristics, the nCHA/PEO/CS-11 scaffold is promising in bone tissue engineering.

## Abstract 2

In this work, we developed a novel porous bone scaffold based on cuttlefish bone-derived nano-carbonated hydroxyapatite reinforced with polyethylene oxide/chitosan fibrous network (nCHA/PEO/CS), which demonstrated enhanced cytocompatibility.

## Graphical abstract

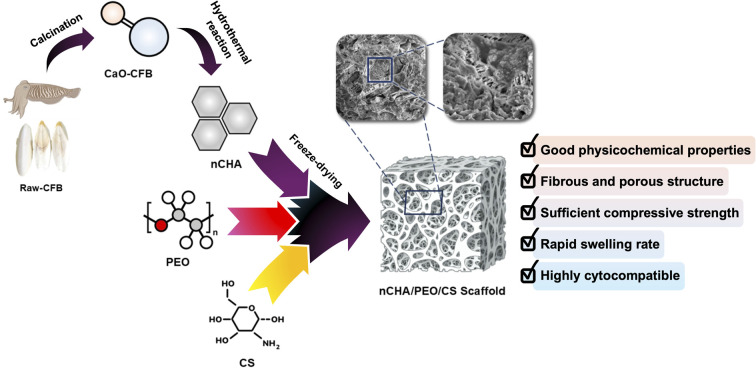

## 1. Introduction

The world's median age is growing due to diminished fertility and increased life expectancy.^1^ The aging of the global population raises the incidence of osteoporosis and related fragility fractures, considerably influencing patient quality of life.^2,3^ Unfortunately, massive bone defects remain a challenge to be treated. Such autografts or xenografts have been widely considered the gold standard for treating bone defects.^4^ However, autografts caused several problems, such as inadequate availability, donor site morbidity, and prolonged surgery, leading to failure of bone replacement.^5^ Furthermore, xenografts can lead to complications, including displacement of the graft materials, foreign body reactions, chronic inflammations, soft-tissue fenestrations, and associated cysts.^6^ Thus, synthetic materials are required to overcome commercial bone graft drawbacks.

Bone consists of an extracellular matrix (ECM) with a fibrous structure arising from the interaction between type-1 collagen and apatite mineral.^7^ Hence, synthetic materials should be reconstructed into a fibrous ECM-like structure with high porosity and surface area.^8,9^ Notably, since ECM in bone possesses multiscale pores, synthetic materials must form a three-dimensional (3D) scaffold that possesses macropores to provide a pathway for cell infiltration into the scaffold and micropores to create surface roughness for governing cell adhesion, proliferation, and differentiation, and enhancing protein adsorption, thereby promoting osteogenesis.^10^ Thus, the fibrous-like and porous structured scaffold, mimicking bone ECM, is desired for favorable regeneration of bone defects.

Various methods, including freeze-drying,^11^ porogen leaching,^12^ 3D printing,^13^ and gas foaming,^14^ have been frequently used to fabricate a 3D scaffold. However, the freeze-drying method is preferable because it can produce a highly porous scaffold with interconnected pores formed by the sublimation of crystallized solvent that leaves a compact structure. The freeze-drying method involves a lyophilization process at a low temperature under vacuum conditions, which prevents contamination and carbon reaction.^15^ The freeze-dried scaffold also exhibits a unique pore structure, such as a fibrous-like, open-cell, and lamellar structure. These pore structures can mimic the native ECM structure that provides suitable curvatures for osteoblastic cell adhesion and migration, thereby enhancing osteogenesis and angiogenesis.^16^ Thus, the freeze-drying method is promising for developing a scaffold with suitable porous structures for bone regeneration.

In bone tissue engineering, synthetic polymers are commonly used as matrices or reinforcers when fabricating porous scaffolds.^12,13,16,17^ Polyethylene oxide (PEO) is one of the synthetic polymers clinically used in biomedical applications due to its biocompatibility and hydrophilicity.^18^ PEO has also been proven for its controllable biomechanical and biodegradable properties.^19^ PEO-based scaffolds can form a fibrous-like structure biomimetic to the native bone ECM.^20^ Since natural bone comprises collagenous ECM, using PEO alone as a scaffold base causes a restriction in bone repair. Hence, the scaffold must have a chemical structure similar to collagen in bone. However, shortcomings of collagen, including poor mechanical properties, high degradability, and lack of osteoconductivity, have been the limitations in clinical applications.^21^ To resolve the lack of type-1 collagen, chitosan (CS) is the best candidate due to its biocompatible, biodegradable, and osteoconductive properties with almost all tissue in the body.^22,23^ The combination of PEO and CS will produce a scaffold with controllable biodegradability, high mechanical strength, and suitable structure to promote bone tissue formation. However, bone comprises 60% apatite mineral, a natural calcium phosphate. Therefore, scaffold reconstruction using calcium phosphate-type materials, while reinforced with PEO/CS to enhance the scaffold integrity, is a necessity for favorable regeneration of bone tissue.^24^

Hydroxyapatite (HA; Ca_10_(PO_4_)_6_(OH)_2_) is a calcium phosphate that has been commonly employed along with polymeric composite due to its biocompatibility and osteoinductivity.^25–27^ The brittleness of HA can increase the biodegradability of polymer-based composites.^28^ Despite the osteogenic and osteoconductive properties of HA, its high stability has been a drawback due to its slow material resorption, which prevents synchronizing with bone ingrowth rate.^29,30^ Since biological bone apatite consists of 2–9 wt% carbonate content,^31^ carbonate content has been a prominent factor in the solubility of apatite under biological conditions.^32,33^ The higher content of carbonate ions in the apatite crystal causes a higher solubility, which leads to a higher resorption rate.^34^ Carbonated hydroxyapatite (CHA; Ca_10−a_(PO_4_)_6−b_(CO_3_)_c_(OH)_2−d_)) is another calcium phosphate that consists of high carbonate content in its apatite crystal, thereby demonstrating a high material resorption for a faster bone formation.^35–37^ Hence, CHA is preferably used due to its efficacy in bone regeneration.

Biogenic-derived calcium (Ca) sources have been quietly used in synthesizing CHA. Biogenic materials contain low concentrations of essential trace elements, which can enhance the properties of CHA. In this study, cuttlefish bone (CFB, *Sepia officinalis*) is used to synthesize CHA. CFB consists of aragonite, a polymorph of calcium carbonate (CaCO_3_) crystal, thus highly comprising Ca.^38^ CFB also lowly contains magnesium (Mg), sodium (Na), and strontium (Sr), which, when turned into CHA, have a significant effect on the bone healing process.^38,39^ Apart from its economic effectiveness, ecological friendliness, and wide availability, CHA based on CFB has been reported for its biocompatibility.^40^

Several methods for synthesizing CHA, including hydrothermal,^41^ co-precipitation,^42^ sol–gel,^43^ nano-emulsion,^44^ and mechanochemical methods,^45^ have been extensively studied. However, the hydrothermal method is a simple method that can produce nanosize-shaped CHA particles. This hydrothermal method involves a hydrothermally aging treatment at high temperatures, typically beyond the boiling point of water, within an autoclave.^46^ During the hydrothermal reaction, the nanocrystalline growth is highly influenced by the dissolution rate of materials, which can be controlled by adjusting the hydrothermal temperature.^47,48^ Therefore, in this study, the hydrothermal process is the preferred method for synthesizing CHA nanoparticles (nCHA) due to its ability to obtain an appropriate size and shape, resulting in a high surface area to volume ratio and aspect ratio of nCHA particles.^49^ nCHA has been reported for its superiority in osteoconduction and osteointegration processes in bone tissue repair.^50,51^

This study investigated the fabrication of a porous scaffold based on nCHA reinforced with a fibrous-like structured PEO/CS network for mimicking bone structure. The nCHA was synthesized using the hydrothermal method to mimic the apatite structure of bone, and CFB was used as a precursor. The nCHA/PEO/CS scaffolds were fabricated by varying the PEO/CS concentration. The effect elucidated by the PEO/CS incorporation was evaluated in terms of chemical-compositional, structural, mechanical properties, and swelling behavior. The *in vitro* cytocompatibility tests in terms of cell viability, adhesion, and osteogenic activity were conducted to determine the most desirable scaffold for bone regeneration.

## 2. Materials and methods

### 2.1. Materials

The CFB used as raw materials (raw-CFB) for the Ca source was purchased from the local fish market in Bandung, Indonesia. Diammonium hydrogen phosphate ((NH_4_)_2_HPO_4_) and ammonium bicarbonate (NH_4_HCO_3_) used as phosphate and carbonate sources, respectively, were purchased from Merck (USA). Ammonium hydroxide (NH_4_OH) 25% solution used for controlling pH was purchased from Merck (USA). PEO with a molecular weight of 400 000 and CS with a medium molecular weight were purchased from Sigma-Aldrich (USA). Acetic acid 100% was purchased from Merck (Germany). Phosphate-buffered saline (PBS) was purchased from Sigma-Aldrich (USA).

### 2.2. Methods

#### 2.2.1. Preparation of CFB-derived calcium oxide (CaO-CFB)

**Fig. 1:**
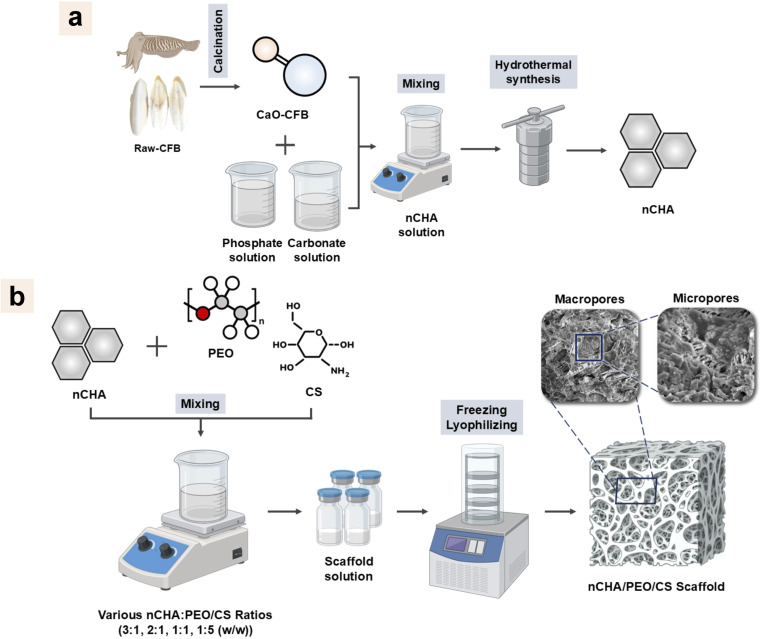
Schematic methods for (a) the nCHA synthesis and (b) nCHA/PEO/CS scaffold fabrication.

The raw-CFB was first rinsed with distilled water and dried. After removing the dorsal shield parts, the lamellar parts were lancet-cut into small chunks.^52^ The raw-CFB was then dried in an oven (Memmert, UN55, Germany) at 100 °C for 6 h to remove organic components. The raw-CFB was crushed using a laboratory disc mill (Kawasaki, T-100, Kobe, Japan), resulting in a powder with reduced particle sizes. The physicochemical properties of the raw-CFB were analyzed using fourier transform infrared spectroscopy (FTIR), X-ray diffraction (XRD), and scanning electron microscopy (SEM). The raw-CFB powder was then calcined in a furnace at 1000 °C for 6 h to obtain CFB-derived calcium oxide (CaO-CFB) (Fig. 1a). For comparative study, the calcination treatment was also carried out at 600 °C for 6 h to confirm the conversion of raw-CFB into CFB-derived calcite (CC-CFB). The CC-CFB and CaO-CFB were examined for their physicochemical properties using FTIR, XRD, and SEM.

#### 2.2.2. Synthesis of nano-carbonated hydroxyapatite (nCHA)

The CaO-CFB (6.048 g) was hydrated to obtain a Ca(OH)_2_ suspension by dispersing it into 60 mL of distilled water and stirring at 350 rpm using a magnetic stirrer for 1 h at 37 °C. Simultaneously, 8.5536 g of (NH_4_)_2_HPO_4_ was added to 70 mL of distilled water, and 5.1192 g of NH_4_HCO_3_ was also added to 50 mL of distilled water, which were separately stirred with a 350 rpm stirring velocity for 30 min at 37 °C. The carbonate solution was titrated dropwise into the phosphate solution at 1 mL min^−1^, followed by 20 min of stirring at 37 °C. The mixture was then titrated into the calcium suspension and then stirred for 1 h at 60 °C. The NH_4_OH solution was added to the mixture to control its pH above 10. The mixture was then hydrothermally treated in a Teflon vessel within an autoclave and heated in the oven for 24 h at 200 °C. The suspension was centrifuged at 4000 rpm for 5 min to obtain material precipitate, which was the nCHA material. The nCHA precipitate was then heated for 24 h at 80 °C. Pulverizing and milling the nCHA block yielded an nCHA powder (Fig. 1a). The nCHA powder was then characterized using FTIR, XRD, transmission electron microscopy (TEM), field emission-scanning electron microscopy (FE-SEM), and energy dispersive X-ray spectroscopy (EDS).

#### 2.2.3. Fabrication of the nCHA/PEO/CS scaffolds

Briefly, PEO was slowly dissolved in 20 mL of 1% (v/v) acetic acid solution and stirred at 350 rpm for 1 h at 70 °C. The CS was then added to the PEO solution with preserved homogeneity and continuously stirred for 1 h. Subsequently, the nCHA was slowly added into the PEO/CS solution and stirred for 1 h. The scaffold compositions varied with the increase in the PEO/CS mixture concentration in the total weight of the scaffold, estimated as 2 g, and the ratio of PEO : CS was controlled at 7 : 3 (Table 1). The suspension was immediately stored in a deep freezer overnight at −40 °C before being lyophilized for 48 h at −55 °C to obtain 3D porous scaffolds (Fig. 1b). These scaffolds were characterized to assess their chemical-compositional, structural, mechanical properties, and swelling behavior.

**Table 1:** Composition of the nCHA/PEO/CS scaffolds

No.	Weight fraction (%)		nCHA : PEO/CS^a^ (w/w)	Sample code
	nCHA	PEO/CS		
1	75.0	25.0	3 : 1	nCHA/PEO/CS-31
2	66.7	33.3	2 : 1	nCHA/PEO/CS-21
3	50.0	50.0	1 : 1	nCHA/PEO/CS-11
4	16.7	83.3	1 : 5	nCHA/PEO/CS-15

a PEO : CS = 7 : 3.

### 2.3. Characterizations

#### 2.3.1. Crystallography analysis

The X-ray diffraction (XRD, PANanalytical, Type X'Pert Pro, Japan) was used to determine the crystallographic properties of the raw-CFB, CC-CFB, CaO-CFB, nCHA, and nCHA/PEO/CS scaffolds. The XRD patterns were recorded in the range of 2*θ*: 10–90° using Cu Kα radiation with *λ* = 0.154 nm. The XRD patterns were identified using data from the Joint Committee on Powder Diffraction Standards (JCPDS). The analyses regarding the lattice parameters, crystallite size, microstrain, and crystallinity of the raw-CFB, CC-CFB, CaO-CFB, nCHA, and nCHA/PEO/CS scaffolds were conducted subsequently.

#### 2.3.2. Functional group analysis

The functional groups within the raw-CFB, CC-CFB, CaO-CFB, nCHA, and nCHA/PEO/CS scaffolds were identified by Fourier transform infrared spectroscopy (FTIR, Thermofisher, Nicolet iS 10, Japan). The FTIR spectra were analyzed in the range of 550–4000 cm^−1^. For the FTIR spectrum of nCHA, the nCHA was mixed with potassium bromide (KBr) and compacted into a pellet, and the nCHA spectrum was observed within the range 400–4000 cm^−1^.

#### 2.3.3. Morphological analysis

The morphologies of the raw-CFB, CC-CFB, CaO-CFB, and nCHA/PEO/CS scaffolds were observed using scanning electron microscopy (SEM, Jeol JSM-6510LA-1400, Japan). For nCHA, transmission electron microscopy (TEM, Jeol, JEM-1400, Japan) and FE-SEM (Jeol, JSM-IT700HR, Japan) were used to observe the morphology of the nCHA particle at a nanoscale. Coupled with FE-SEM, the elemental analysis related to the carbon (C), calcium (Ca), oxygen (O), and phosphorus (P) content within the nCHA was conducted using energy dispersive X-ray spectroscopy (EDS). The acquired atomic masses were utilized to calculate the molar ratio of Ca to P (Ca/P) and carbonate (CO_3_) content within the nCHA. The nCHA particle size was determined using ImageJ software by analyzing numerous randomly selected particles. For the nCHA/PEO/CS scaffolds' SEM results, the macro- and micropore sizes of the scaffold were also calculated using ImageJ, and the macro- and microporosity of the scaffolds were analyzed using Origin.

#### 2.3.4. Mechanical properties analysis

The universal testing machine (UTM, IMADA ZTA-1000N, Japan) was used to determine the compressive strength of the nCHA/PEO/CS scaffolds. The scaffolds were prepared with a dimension of 1 × 1 × 0.2 cm^3^ and then tested at 60 mm min^−1^ compressive speed rate. The compression stopped at the breaking point of the scaffolds. The compressive strength value was measured by the formula as follows in eqn (1):1
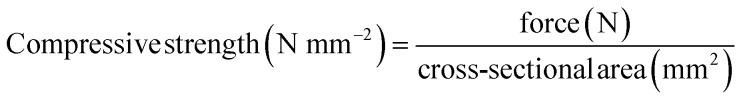


These compressive test procedures were repeated four times for each group (*n* = 4).

#### 2.3.5. Swelling behavior analysis

The gravimetric method was used to assess the swelling behavior of the scaffolds in terms of liquid absorption.^53^ The scaffolds were prepared with a dimension of 1 × 1 × 1 cm^3^ and then weighed at dried condition (*W*_d_). The scaffolds were soaked in a PBS medium with a controlled pH 7.4 and incubated at 37 °C. At the prescribed intervals, the wet scaffolds were removed from the PBS medium and gently dipped in filter paper to eliminate excess liquid on their surfaces. The scaffolds in wet condition were then weighed (*W*_w_). The swelling test was conducted with 70 min as the maximum swelling time, and the swelling ratio in each 10 min time increment was estimated by the formula as follows in eqn (2):2
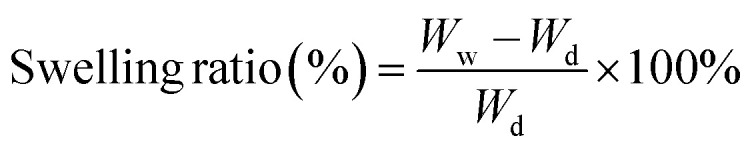


when the swelling ratio approached maximum, the swelling test procedures were repeated thrice for each group (*n* = 3).

#### 2.3.6. Cell culture

MC3T3E1 cells (European Collection of Authenticated Cell Cultures (ECACC) No. 99072810, UK) were cultured in MEM-α medium (Gibco, USA) with 10% fetal bovine serum (FBS, Gibco, CA, USA), 2% penicillin–streptomycin (Gibco, CA, USA), and 0.5% fungizone (Gibco, USA). The MC3T3E1 cells were seeded at the bottom of 96-well plates at a density of 2 × 10^4^ cells per well. The MC3T3E1 cells were incubated at 37 °C in 5% CO_2_ for 24 h. The cell growth medium was replaced every 3 days until approaching confluence. The confluent cells were harvested using 0.25% trypsin-ethylenediaminetetraacetic acid (EDTA, Gibco, USA), and after being centrifugated, the cells were seeded into a new culture dish. The above procedures were conducted repeatedly until sufficient amounts of cells were obtained for each *in vitro* test.

#### 2.3.7. MTT assay

Briefly, MC3T3E1 cells were seeded in a 96-well plate at a density of 2 × 10^4^ cells per well (*n* = 3). Prior to cell seeding, the scaffolds were dispersed in 40 mL of distilled water until becoming homogeneous suspension and diluted at serially diluted concentrations of 1000, 500, 250, 125, 62.5, 31.3, 15.6, and 7.81 μg mL^−1^. Next, 100 μL of the scaffold suspension was added to the cells in each well, with wells without scaffold suspension addition being considered a control, then incubated at 37 °C in 5% CO_2_ for 24 h.

The MTT assay was used to assess the cytocompatibility of the scaffold against MC3T3E1 cells.^27^ The cell viability was evaluated through the cell metabolic activity after 24 h of incubation. Briefly, the medium was gently aspirated from each well. Next, 100 μL of MTT solution (Biobasic, USA) with a concentration of 0.5 mg mL^−1^ was added to the well and incubated for 4 h. Then, 100 μL of dimethyl sulfoxide (DMSO, Merck, Germany) was added to each well. The absorbance at 570 nm was measured using a Tecan Spark® analyzer (Tecan, Switzerland). The cell viability was assessed by measuring the absorption value of the test cultures, expressed as a percentage of absorption for unstimulated control cultures.^54^ The cell viability was determined by the formula as follows in eqn (3):3
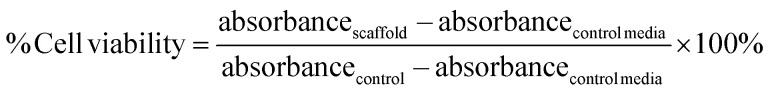


The half maximum inhibitory concentration (IC_50_) quantified the inhibitor amount required to suppress biological processes or components by 50%. Examining the IC_50_ determines the scaffold's safe dose to avoid cell growth inhibition. The IC_50_ value was analyzed using non-linear curve fitting.

#### 2.3.8. Cell adhesion

In brief, MC3T3E1 cells were seeded on the scaffold surfaces for cell adhesion in a 24-well plate with a density of 5 × 10^3^ cells per well (*n* = 3) for 48 h. After incubation, The growth medium was aspirated from each well, and then the scaffolds were rinsed with PBS to discard unadhered cells on the scaffold surfaces. To fix the cells, the washed cells were incubated in a 4% paraformaldehyde solution for 2 h at 4 °C. For SEM observation, the scaffolds were dehydrated with graded ethanol solutions (20, 30, 40, 50, 60, 70, and 100%), followed by drying overnight. After obtaining the SEM images, the cell spreading area was analyzed to quantify cell adhesion. For immunofluorescence staining, the intracellular F-actin cytoskeleton of cells was stained with phalloidin, and the cell nucleus was counterstained with DAPI for 5 min, followed by rinsing with PBS thrice. The cell morphology was observed using confocal laser scanning microscopy.

#### 2.3.9. Alkaline phosphatase (ALP) activity

MC3T3-E1 cells were seeded on the scaffold in a 24-well plate at a density of 1 × 10^4^ cells per well (*n* = 3). The cells were cultured with the scaffolds in a differentiation medium, the growth medium supplemented with β-glycerol phosphate (1 × 10^−2^ M) and ascorbic acid (50 μg mL^−1^). Every 2 days, the culture medium was changed. After 7 days, the cells were rinsed with PBS thrice and lysed using cell lysis buffer. The ALP activity was then assessed using an ALP assay kit and normalized against the total protein concentration of the scaffold determined using a protein assay kit.

#### 2.3.10. Statistical analysis

The compressive strength, swelling ratio, cell spreading area, and ALP activity were analyzed using a one-way analysis of variance (ANOVA), with Tukey's multiple comparison test used for evaluating the difference between groups. The cell viability data was analyzed using a two-way ANOVA and Tukey's multiple comparisons test. The *p* < 0.01 and *p* < 0.001 were considered statistically significant, and each data was presented as mean ± standard deviation (SD).

## 3. Results and discussion

### 3.1. Properties of raw materials

The raw-CFB was first decomposed into CaO-CFB by calcination before being used as the Ca source in the nCHA synthesis. The heat energy received by CaCO_3_ polymorphic crystals in the raw CFB caused them to move faster and break their chemical bonds, causing the decomposition reaction to form CaO crystals, as follows in eqn (4). The endothermic transformation of CaCO_3_ to CaO phase commonly occurs at above 800 °C.^55^

4 CaCO_3(s)_ → CaO_s_ + CO_2(g)_

**Fig. 2:**
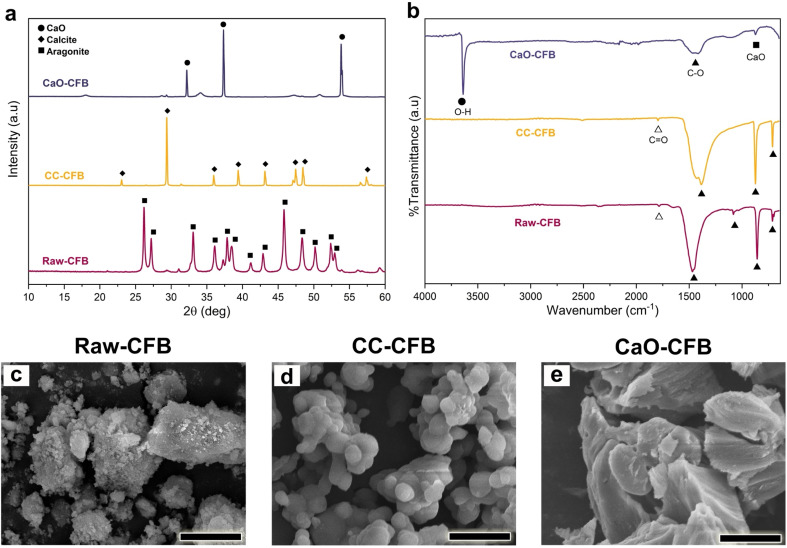
(a) XRD patterns and (b) FTIR spectra of the raw-CFB, CC-CFB, and CaO-CFB. (c–e) Morphologies of the raw-CFB, CC-CFB, and CaO-CFB. Scale bars: 5 μm (c–e).

The XRD patterns of the raw-CFB, CC-CFB, and CaO-CFB showed differences in the crystal structure (Fig. 2a). The XRD pattern of the raw-CFB corresponded to aragonite (PDF number 901-6527), which strongly exhibited at diffraction angles (2*θ*) of 26.2° and 45.8°. The CC-CFB diffraction pattern indicated peaks corresponding to calcite (PDF number 47-1743) that strongly exhibited at a 2*θ* of 29.4°. The unit cell volume, crystallite size, and crystallinity of the CC-CFB were higher than those of the raw-CFB (Table 2). Although the calcination at 600 °C caused a conversion of aragonite polymorphic crystal into calcite crystal, calcite has a highly stable phase, thereby unsuitable for using CC-CFB as an nCHA precursor. Contrarily, the CaO-CFB diffraction pattern showed diffraction peaks corresponding to CaO (PDF number 37-1497) with a main diffraction peak at 2*θ* of 37.4° and 53.8°. Although the unit cell volume of the CaO-CFB was lower than that of raw-CFB, the crystallite size and crystallinity of the CaO-CFB were higher than those of the raw-CFB (Table 2). These results suggest that at 1000 °C, the aragonite polymorphic crystal was decomposed into CaO crystal. CaO crystal is reportedly less stable than calcite crystal. Hence, CaO-CFB was more suitable as a precursor in synthesizing nCHA.

**Table 2:** Crystallographic properties of the raw-CFB, CC-CFB, CaO-CFB, and nCHA

No.	Sample	Lattice parameter (Å)			Unit cell volume (cm^3^) (10^−22^)	Crystallite size (*s* ± Δ*s*) (nm)	Microstrain (*ε*) (10^−3^)	Degree of crystallinity (%)
		*a* = *b*	*c*	*a*/*c*				
1	Raw-CFB	5.752, 4.972^a^	7.936	—	2.26	58.4 ± 3.41	1.55 ± 0.66	82.2
2	CC-CFB	4.989	17.08	—	3.68	82.5 ± 2.24	1.09 ± 0.72	91.8
3	CaO-CFB	4.813^b^		—	1.11	70.9 ± 6.24	2.33 ± 0.61	84.6
4	nCHA	9.433	6.892	0.730	5.31	56.7 ± 2.74	1.85 ± 0.14	79.9

a ≠ *b*.

b *a* = *b* = *c*.

The FTIR spectra of the raw-CFB, CC-CFB, and CaO-CFB showed transformations in functional groups (Fig. 2b). The raw-CFB and CC-CFB spectra displayed the main vibrational bonds of aragonite and calcite, respectively, which were the C=O and C–O bonds observed at 1784 and 1471–715 cm^−1^, respectively. In the CaO-CFB spectrum, the CaO bond was observed at 871 cm^−1^, which appeared as a consequence of a severe reduction in the C–O bond within the raw-CFB, thus suggesting that the aragonite in the raw-CFB was decomposed into CaO and released carbon dioxide (CO_2_) by calcination at 1000 °C. Although, in the CaO-CFB spectrum, the O–H stretching band appeared at 3637 cm^−1^ due to the hydration process of CaO,^26^ the diffraction data clarified that the CaO-CFB formed a CaO crystal structure.

The morphologies of the raw-CFB, CC-CFB, and CaO-CFB showed changes in gross structure (Fig. 2c–e). The raw-CFB had a tiny irregular-shaped particle in its gross surface, creating a rough and brittle structure (Fig. 2c). The gross surface of the CC-CFB was more delicate than that of the raw-CFB with grown particles (Fig. 2d). These results suggest that the calcination at 600 °C caused the nucleation of aragonite polymorphic crystal that crystallized into calcite crystal to form a fine structure and agglomerated spherical-shaped particles. Although the CaO-CFB also exhibited delicate surfaces, it had a bulk-chunked structure (Fig. 2e), thus suggesting that the particle agglomeration in the time increment under 1000 °C occurred quickly before decomposing into the CaO phase. Hence, the structure of CaO-CFB indicated a stable phase, which was more appropriate as an nCHA precursor.

### 3.2. Properties of the nCHA based on CFB

**Fig. 3:**
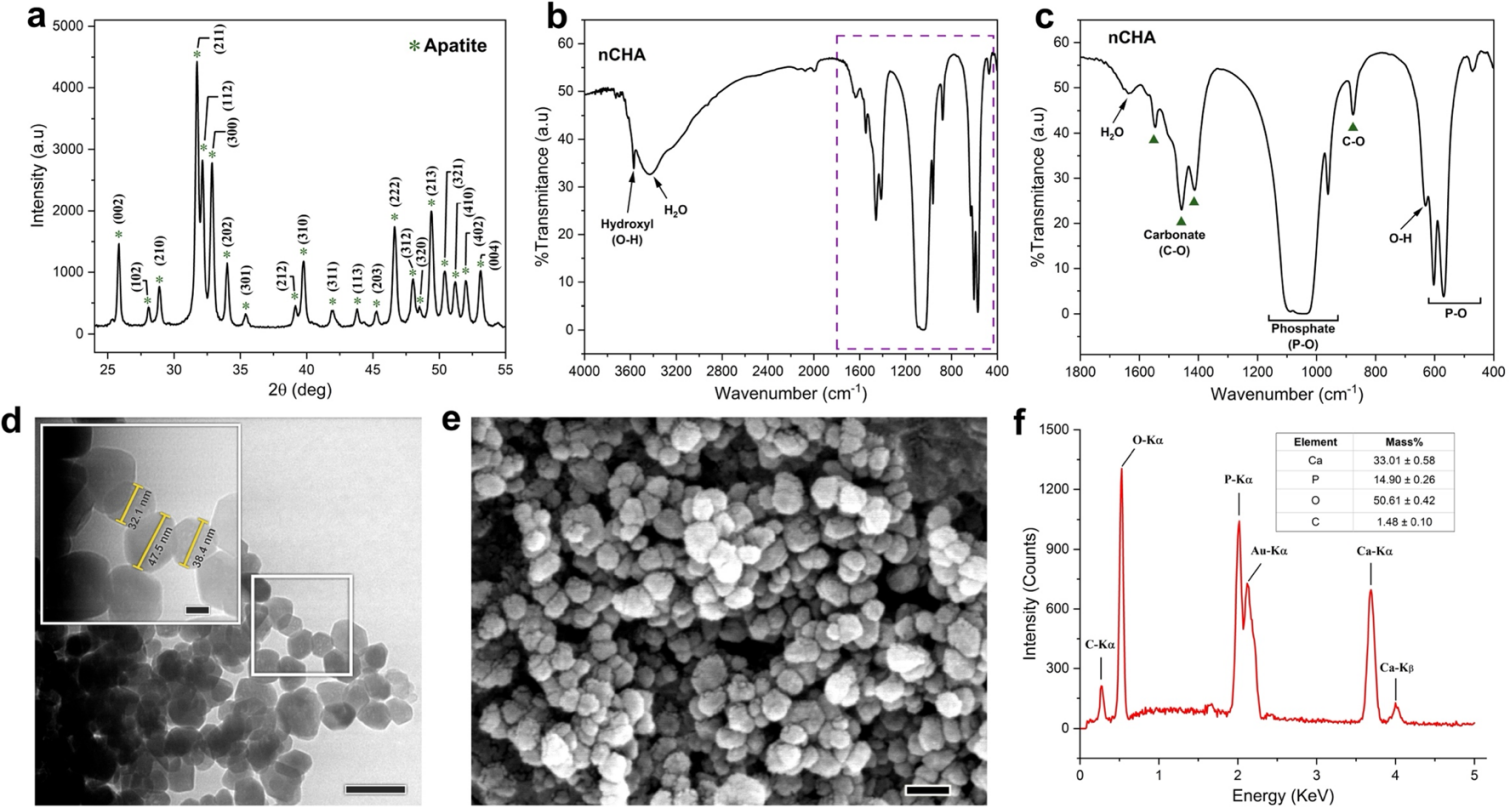
(a) XRD pattern, (b) FTIR spectrum, and (c) magnified FTIR spectrum of nCHA. (d) TEM, (e) FE-SEM images and (f) EDS spectrum of nCHA. Scale bars: 100 nm and 20 nm (d), and 100 nm (e).

The XRD pattern of the nCHA exhibited distinct peaks corresponding to apatite (PDF number 09-0432), with the main diffraction peaks observed at 2*θ* of 31.7°, 32.1°, 32.9°, and 34.0° attributed to the hexagonal lattice planes of (211), (112), (300), and (202), respectively (Fig. 3a). No secondary phases were detected in the nCHA diffraction pattern, thus suggesting that the nCHA had a high purity of apatite phase and avoided impurities. Both lattice parameters of *a* and *c* in the nCHA were larger than those of typically HA (*a* = 9.184 and *c* = 6.884 Å), respectively (Table 2). In general, A-type CHA forms due to CO_3_^2−^ substitution to the OH^−^ site that induces contraction of the *a*-axis and expansion of the *c*-axis in the apatite lattice plane, while, in the B-type CHA, CO_3_^2−^ substitutes the PO_4_^3−^ site that does *vice versa* to the apatite lattice plane. The lattice parameters *a* and *c* of the nCHA showed that both the *a*- and *c*-axis were expanded, thus indicating the simultaneous substitution of CO_3_^2−^ to both the OH^−^ and PO_4_^3−^ sites in the apatite lattice plane.^56^ These results suggest that the nCHA formed AB-type CHA.

The FTIR spectrum of the nCHA showed the main characteristic bands of CHA (Fig. 3b and c). The peaks corresponding to the *ν*_3_ phosphate (P–O) asymmetric stretching, *ν*_1_ P–O stretching, *ν*_4_ P–O asymmetric bending, and *ν*_2_ P–O stretching bands were observed at 1094–1031, 961, 604–572, and 472 cm^−1^, respectively. The slight peaks observed at 3571 and 631 cm^−1^ corresponded to the O–H bending and stretching bands. The broad peaks at 3442 and 1639 cm^−1^ corresponded to the H_2_O absorption band. The carbonate (C–O) absorption bands were observed at 1546–876 cm^−1^. The peak at 1546 and 1457 cm^−1^ corresponded to the *ν*_3_ C–O bending band attributed to the CO_3_^2−^ substitution in the A-site (OH^−^) and B-site (PO_4_^3−^), respectively. Previous studies pointed out that the *ν*_2_ C–O bending band at 876 cm^−1^ and the *ν*_3_ C–O stretching band at 1414 cm^−1^ were also attributed to A- and B-sites substitution, respectively. The intensity ratio between peaks at 1546 and 1414 cm^−1^ clearly showed the ratio of A-type to B-type CO_3_ content, which estimated that the peak intensity of B-type CO_3_ was higher than that of A-type CO_3_.^30,36,56^ These FTIR results deal with the XRD data, showing that the nCHA formed an AB-type CHA.

The morphologies of the nCHA clearly showed nanoparticles with rounded hexagonal shapes (Fig. 3d and e). The nanoparticle size of the nCHA was in the range of 32–76 nm (Fig. 3d and e), which is approximately close to the nanoparticle size of bone apatite.^57^ The nCHA particle size was ascribed to the nCHA crystallite size by diffraction analysis (Table 2). The nanosize of nCHA may provide a high surface area for ion exchange and interaction with cells and proteins. The elemental analysis revealed that the Ca/P molar ratio was 1.712, which is very close to the Ca/P molar ratio of biological apatite 1.71.^27^ The CO_3_ content amount within the nCHA was 7.38 wt%, which is considerably within the typical range of CO_3_ content in bone apatite, 2–9 wt%.^57^ Hence, the high CO_3_ content within the nCHA may promote a high material resorption.^34^

### 3.3. Properties of the nCHA/PEO/CS scaffolds

#### 3.3.1. Physicochemical characteristics of the nCHA/PEO/CS scaffolds

**Fig. 4:**
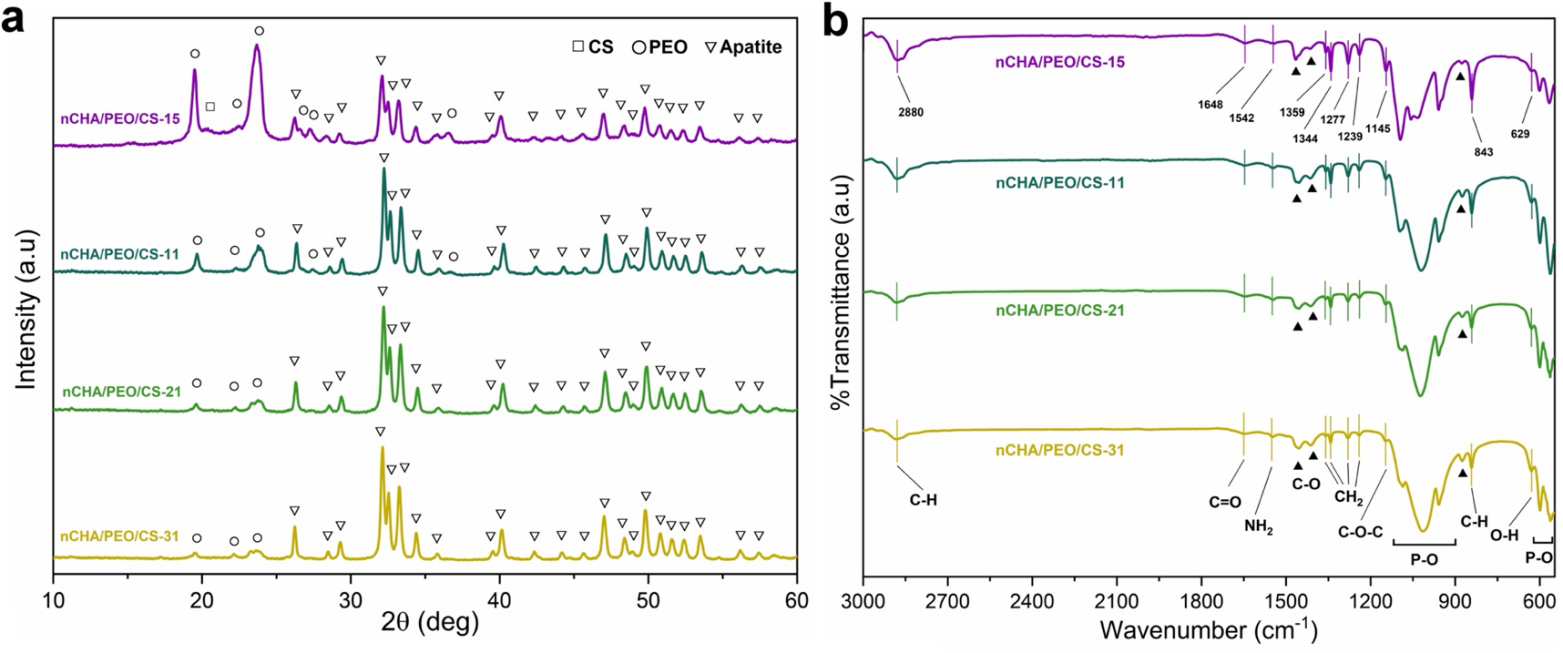
(a) XRD patterns and (b) FTIR spectra of the nCHA/PEO/CS scaffolds.

The XRD patterns of the nCHA/PEO/CS scaffolds showed differences in diffraction peaks (Fig. 4a). In the nCHA/PEO/CS-31 diffraction pattern, the diffraction peaks corresponded to PEO (PDF number 50-2158) appeared that weakly exhibited at 19.5° and 23.7° attributed to the (120) and (112) helix lattice planes of the crystallized PEO chains. In the nCHA/PEO/CS-21 and nCHA/PEO/CS-11 diffraction patterns, the PEO peak intensity increased by increasing the PEO/CS concentration. In the nCHA/PEO/CS-15 diffraction pattern, a slight diffraction peak at 20.3° corresponded to CS (PDF number 39-1894) was clearly shown, attributing to the (200) helix lattice plane of the crystallized chitosan chains. The increase in the PEO/CS concentration caused the decrease in the nCHA/PEO/CS scaffold crystallinity, causing it to be lower than the nCHA crystallinity due to the presence of low crystalline PEO and CS in the scaffold (Table 3). The nCHA/PEO/CS-11 and nCHA/PEO/CS-15 had the lowest crystallinity (Table 3). However, the nCHA/PEO/CS-15 diffraction pattern showed that the main peak intensity of the PEO (112) was higher than that of the apatite (211), suggesting that the concentration of PEO was higher than that of nCHA. Compared to the nCHA/PEO/CS-11 diffraction pattern, the peak intensity difference between nCHA and PEO showed *vice versa*, indicating the higher concentration of nCHA. Consequently, the nCHA/PEO/CS-15 contained a smaller amount of the nCHA than the nCHA/PEO/CS-11, which may decrease the osteoconductivity of the scaffold. Hence, the nCHA/PEO/CS-11 had suitable crystallographic properties. The low crystallinity of the nCHA/PEO/CS-11 scaffold may promote an appropriate biodegradability rate that can synchronize with the new bone ingrowth rate. The low crystallinity of the nCHA/PEO/CS-11 scaffold may imply the occurrence of dislocation, which, in turn, enhances cell adhesion, proliferation, and differentiation.^26^

**Table 3:** Crystallinity of the nCHA and nCHA/PEO/CS scaffolds

No.	Sample	Degree of crystallinity (%)
1	nCHA	79.9
2	nCHA/PEO/CS-31	77.4
3	nCHA/PEO/CS-21	72.8
4	nCHA/PEO/CS-11	71.8
5	nCHA/PEO/CS-15	63.2

The FTIR spectra of the nCHA/PEO/CS scaffolds showed differences in functional groups (Fig. 4b). In all scaffolds, the peaks at 2880, 1359–1344, 1277–1239, 1145, and 843 cm^−1^ corresponded to the C–H asymmetric stretching, CH_2_ wagging, CH_2_ twisting, C–O–C stretching, and CH_2_ asymmetric rocking vibrational bands, respectively, which were ascribed to the PEO functional groups.^58^ The slight peaks, corresponding to the C=O stretching and NH_2_ stretching vibrational bands ascribed to the chitosan functional groups, appeared at 1644 and 1542 cm^−1^, respectively.^59^ The appearance of PEO and CS functional groups in the nCHA/PEO/CS spectra indicates that the PEO and CS molecules endured during lyophilization, and only the solvent underwent sublimation.^15^ The nCHA/PEO/CS scaffolds performed the carbonate, phosphate, and hydroxyl bands, thus suggesting that the scaffold contained nCHA. The *ν*_3_ P–O asymmetric stretching, *ν*_1_ P–O stretching, and *ν*_4_ P–O asymmetric bending bands were present at 1096–1020, 957, and 600–562 cm^−1^, respectively. The slight peak at 630 cm^−1^ was ascribed to the O–H bending band. The *ν*_3_ C–O bending and stretching bands remained observed at 1456 and 1412 cm^−1^, with the *ν*_2_ C–O bending band at 874 cm^−1^, thus suggesting that only B-type CO_3_ was detected. As the PEO/CS concentration increased, the peak intensity of PEO and CS functional groups increased, while the apatite functional groups decreased, thus dealing with the XRD data. Although the chemical composition of the nCHA/PEO/CS scaffolds was changed, the remaining CO_3_ content within the scaffold can mimic the chemical composition of bone.

**Fig. 5:**
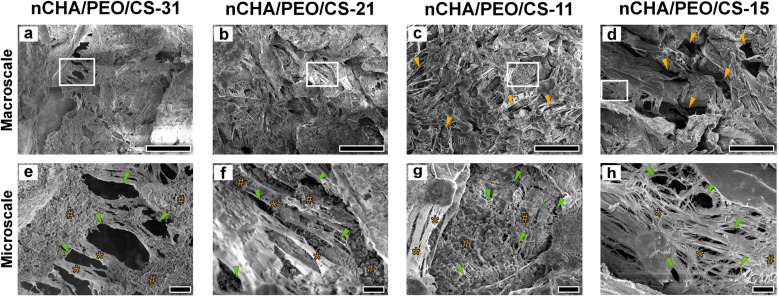
Morphologies of the nCHA/PEO/CS scaffolds in (a–d) macro- and (e–h) microscale. The characters “#,” “*,” orange and green arrowheads indicate nCHA, PEO/CS fibrous network, macropores, and micropores, respectively. Scale bars: 100 μm (a–d) and 10 μm (e–h).

The nCHA/PEO/CS scaffolds clearly showed their macro- and microstructure (Fig. 5). At the macroscale, the nCHA/PEO/CS scaffolds showed a compact and robust structure with heterogeneously and irregularly formed macropores (Fig. 5a–d). These macropores sizes involved in the nCHA/PEO/CS scaffolds were >50 μm and indicated increases in the size of the macropores as the PEO/CS concentration increased (Table 4). The nCHA/PEO/CS-11 and nCHA/PEO/CS-15 scaffolds exhibited macropores size >100 μm (Fig. 5c and d), the ideal macropore size for facilitating cell penetration and blood vessel formation.^60^ Although the nCHA/PEO/CS-11 and nCHA/PEO/CS-15 scaffolds had appropriate micropore sizes for bone regeneration, the nCHA/PEO/CS-15 lack in the nCHA content may turn to the low osteoconductivity. Hence, the nCHA/PEO/CS-11 is desirable in bone regeneration. At the microscale, the nCHA/PEO/CS scaffolds clearly showed the fibrous-like structured PEO/CS network that cross-linked the nCHA (Fig. 5e–h). The fibrous-like structure of the PEO/CS network within the scaffold mimics the fibrous structure of ECM in the bone that may provide a large surface area for cell recruitment and differentiation, thereby promoting endogenous tissue regeneration when filling it into the bone defect.^7,17^ In the nCHA/PEO/CS-15 scaffold, the nCHA particles were seemingly covered by crystallized polymeric chains that formed a dense structure, creating a flat surface curvature (Fig. 5h). On the contrary, in the nCHA/PEO/CS-31, nCHA/PEO/CS-21, and nCHA/PEO/CS-11 scaffolds, the nCHA particles were well-distributed and uncovered that created rough surface curvatures (Fig. 5e–g). The rough surface curvature may improve cell adhesion and migration on the scaffold surface, thereby promoting faster bone ingrowth.^61^ All nCHA/PEO/CS scaffolds involved micropores with size <10 μm that formed among the fibrous structure of PEO/CS that cross-linked nCHA particles (Fig. 5e–h), which indicated increases in the size of the micropores as the PEO/CS concentration increased (Table 4). The micropore size <10 μm can create a rough surface for facilitating cell adhesion, proliferation, and differentiation.^62^ In the nCHA/PEO/CS-31 and nCHA/PEO/CS-15 scaffolds, the micropores were irregular and diversely distributed (Fig. 5e and h). Meanwhile, the micropore distribution was heterogeneous in the nCHA/PEO/CS-21 scaffold (Fig. 5f). In contrast, the nCHA/PEO/CS-11 scaffold had regularly, homogeneously, and uniformly distributed micropores (Fig. 5g). The regular, homogeneous, and uniform micropores in the nCHA/PEO/CS-11 scaffold may encourage cells and cell nutrients to engage in the scaffold.^63^ These results suggest that the nCHA/PEO/CS-11 scaffold formed a fibrous-like and porous structure with suitable pores characteristics and surface curvatures, which may promote favorable bone regeneration.

**Table 4:** Pore sizes and porosities of the nCHA/PEO/CS scaffolds

No.	Sample	Macropore size (>50 μm)	Micropore size (<10 μm)	Macroporosity (%)	Microporosity (%)
1	nCHA/PEO/CS-31	56.9 ± 8.96	5.51 ± 1.20	51.3	59.4
2	nCHA/PEO/CS-21	65.8 ± 13.0	2.49 ± 0.61	53.9	61.6
3	nCHA/PEO/CS-11	115 ± 18.2	3.99 ± 0.86	57.6	64.8
4	nCHA/PEO/CS-15	117 ± 24.2	5.60 ± 1.33	62.1	65.1

Coupled with the structural properties of the nCHA/PEO/CS scaffolds, the two-dimensional porosity in terms of macro- and microporosity of the nCHA/PEO/CS scaffolds showed increases with the increase in the PEO/CS concentration (Table 4). The ideal porosity of the scaffold suitable for cell penetration and nutrient perfusion was >60%.^64^ Hence, all nCHA/PEO/CS scaffolds had a microporosity close to 60%. However, only the nCHA/PEO/CS-11 and nCHA/PEO/CS-15 had a macroporosity close to 60%. Considering the most proper structural characteristics of the nCHA/PEO/CS-11 scaffold, the nCHA/PEO/CS-11 was preferable for bone regeneration.

#### 3.3.2. Mechanical properties of the nCHA/PEO/CS scaffolds

**Fig. 6:**
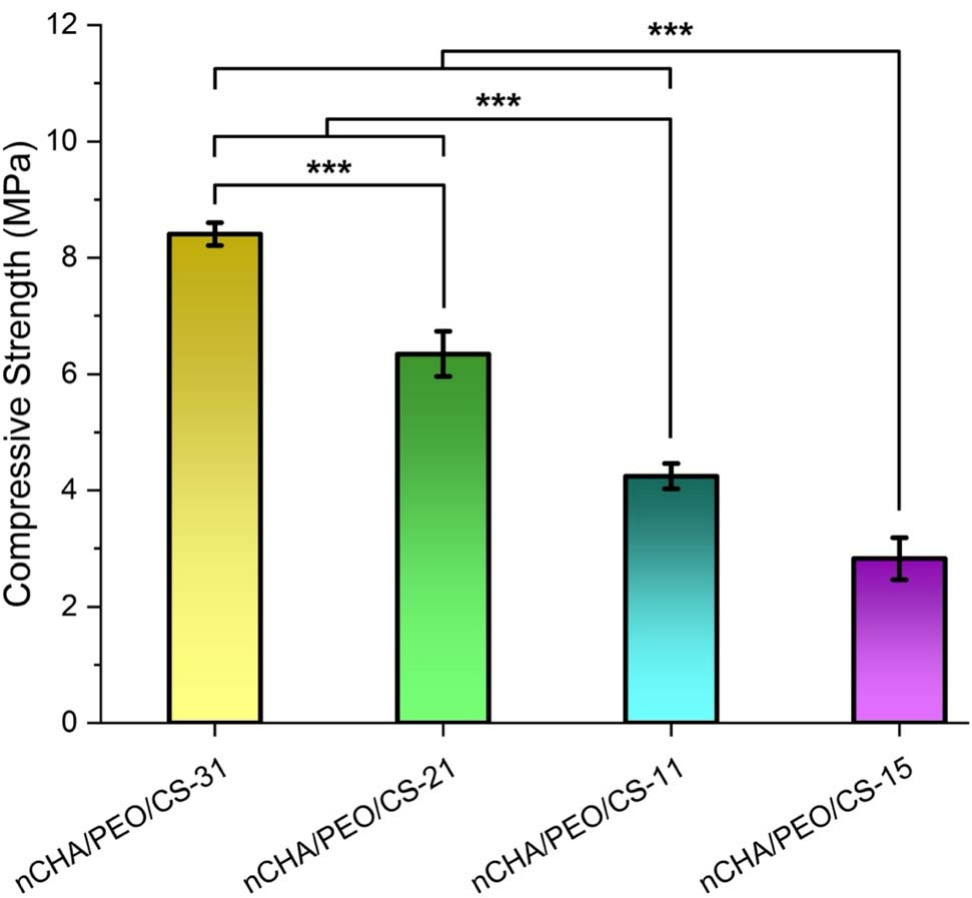
Compressive strengths of the nCHA/PEO/CS scaffolds. ***p < 0.001.

The nCHA/PEO/CS scaffolds demonstrated decreases in compressive strength (Fig. 6). The compressive strengths of the nCHA/PEO/CS-31, nCHA/PEO/CS-21, nCHA/PEO/CS-11, and nCHA/PEO/CS-15 scaffolds were 8.41 ± 0.20, 6.35 ± 0.19, 4.24 ± 0.22, and 2.83 ± 0.36 MPa, respectively. Hence, the compressive strengths of the nCHA/PEO/CS scaffolds significantly decreased due to the increase in the PEO/CS concentration (*p* < 0.001). Nevertheless, these nCHA/PEO/CS scaffolds' compressive strengths precisely resembled the compressive strength of cancellous bone, 2–12 MPa.^57^ The decrease in compressive strength was caused by increased incorporation level due to more concentrated PEO/CS in the scaffold, thus reducing the compactness and robustness of the structure of the composite scaffold. These compressive strength results coincided with the decrease in the macro- and microporosity of the scaffold (Table 4). Virtuing its appropriate porous characteristics, the nCHA/PEO/CS-11 scaffold had sufficient mechanical strength for bone regeneration.

#### 3.3.3. Swelling behavior of the nCHA/PEO/CS scaffolds

**Fig. 7:**
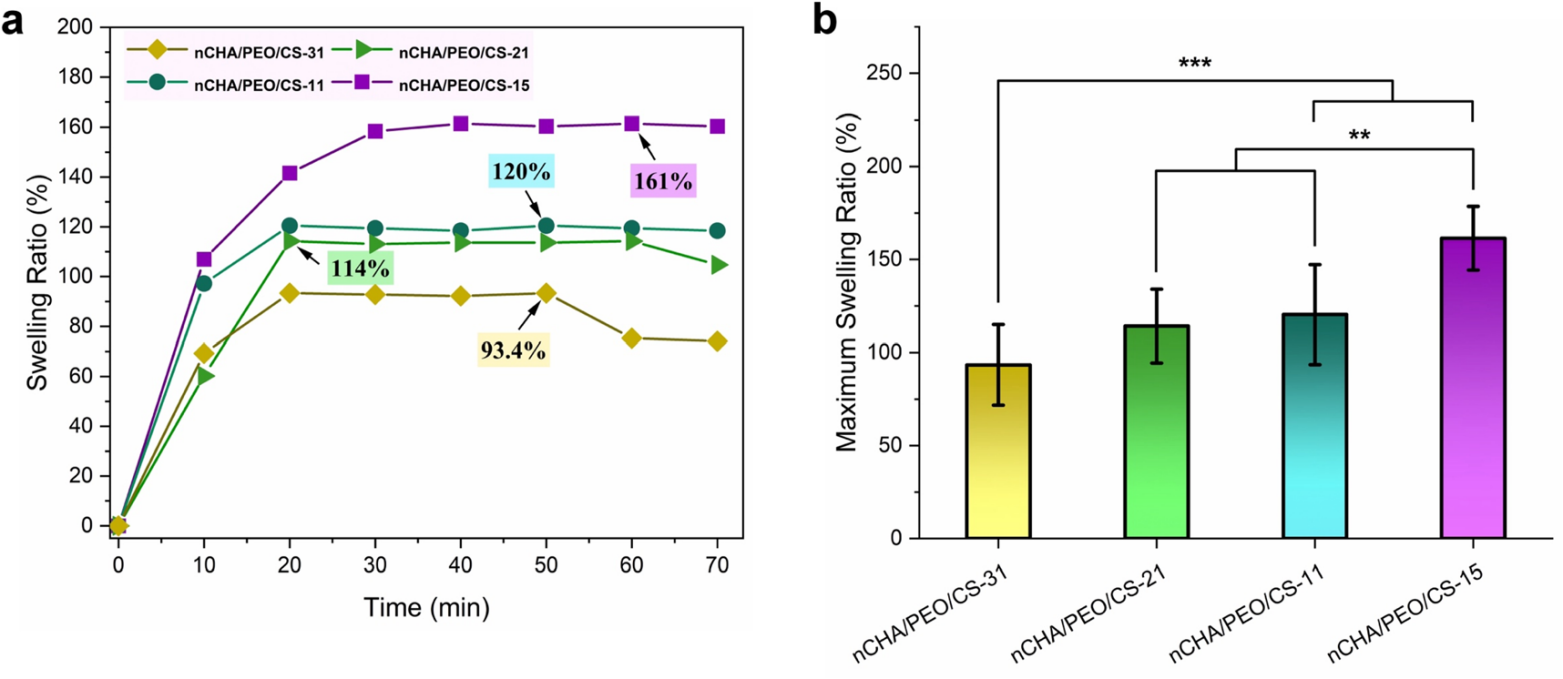
(a) Swelling profiles and (b) maximum swelling ratios of the nCHA/PEO/CS scaffolds. **p < 0.01 and ***p < 0.001.

The nCHA/PEO/CS scaffolds demonstrated changes in swelling behavior that were assessed in a PBS medium with pH 7.4 (Fig. 7a). As the PEO/CS concentration increased, the swelling curve of the nCHA/PEO/CS scaffold showed improvement in the swelling ratio, which coincided with the increase in the macro- and micropore sizes in the nCHA/PEO/CS scaffold (Table 4). The enhanced swelling ability was also related to the induced hydrophilic PEO/CS network within the composite scaffold, correlated to the detected amino (NH_2_) and hydroxyl (O–H) groups in the FTIR results (Fig. 4b), which induced hydrophilicity in uptaking and penetrating the fluidic liquid-phase into the scaffold. In the nCHA/PEO/CS-15 swelling curve, the swelling ratio of the nCHA/PEO/CS-15 scaffold was 161% at 40 min, and it began being saturated. In contrast, the swelling ratios of the nCHA/PEO/CS-31, nCHA/PEO/CS-21, and nCHA/PEO/CS-11 scaffolds were 93.3, 114, and 120%, respectively, at 20 min before being saturated. These results suggest that the nCHA/PEO/CS-31, nCHA/PEO/CS-21, and nCHA/PEO/CS-11 scaffolds had rapid swelling compared with the nCHA/PEO/CS-15 scaffold. The rapid attainment of saturation was caused by progressive cross-linking between the PEO/CS polymeric structure and nCHA particles, which may accelerate liquid infiltration into the scaffold *via* the pore pathway. However, the nCHA/PEO/CS-31 and nCHA/PEO/CS-21 scaffolds exhibited decreases in swelling ratio after 50 and 60 min, respectively, thus indicating a resisted swelling behavior. Contrastingly, the nCHA/PEO/CS-11 scaffold exhibited a constant swelling ratio before 70 min, thus indicating a well-regulated swelling behavior. Since the incorporation of PEO/CS increased the swelling ratio of the scaffold, the nCHA/PEO/CS-11 scaffold showed good swelling behavior with rapid swelling, which may promote penetration of bodily fluids, proteins, and cells into the porous scaffold.^65^

Related to the swelling behavior, the maximum swelling ratio of the nCHA/PEO/CS scaffolds increases with the increase in the PEO/CS concentration (Fig. 7b). The maximum swelling ratios of the nCHA/PEO/CS-31, nCHA/PEO/CS-21, nCHA/PEO/CS-11, and nCHA/PEO/CS-15 scaffolds were 93.4 ± 10.8, 114 ± 19.9, 120 ± 8.45, and 161 ± 20.3%, respectively. Hence, the maximum swelling ratios of the nCHA/PEO/CS scaffolds significantly increased due to the increase in the PEO/CS concentration (*p* < 0.01). These results were cohesive with the enhancement in the swelling ability of the nCHA/PEO/CS as the increase in the PEO/CS incorporation (Fig. 7a). Owing to the suitable swelling behavior and rapid swelling, the nCHA/PEO/CS-11 scaffold showed well-expected swelling properties that may encourage a rapid biodegradability of the scaffold for bone regeneration.^66^

#### 3.3.4. *In vitro* cell viability of the nCHA/PEO/CS-21 and nCHA/PEO/CS-11 scaffolds

The prior analyses showed that the nCHA/PEO/CS-11 scaffold had the best characteristics as a material for bone regeneration. Therefore, the nCHA/PEO/CS-11 scaffold must be tested for its cytocompatibility to assess cell response to the scaffold, which is required for bone formation efficacy. For comparison analysis, the nCHA/PEO/CS-21 scaffold was used as a comparative sample to evaluate the influence of composition within the scaffold on the viability of MC3T3E1 cells.

**Fig. 8:**
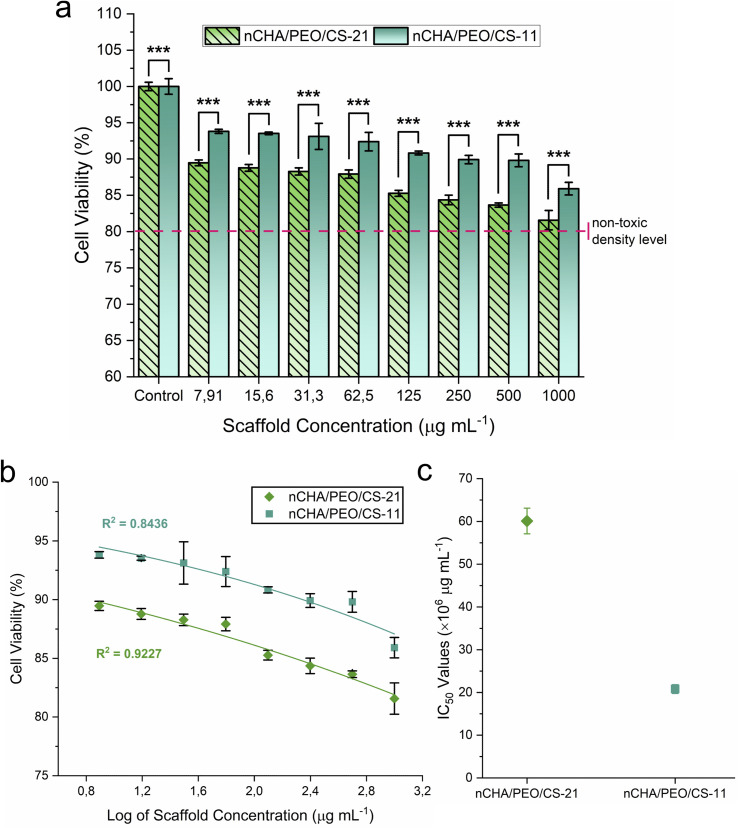
(a) Viabilities of MC3T3E1 cells, (b) IC_50_ analysis by non-linear curve fitting, and (c) IC_50_ values of the nCHA/PEO/CS-21 and nCHA/PEO/CS-11 scaffolds. ***p < 0.001.

The nCHA/PEO/CS-21 and nCHA/PEO/CS-11 scaffolds exhibited increases in cell viability by the low scaffold concentrations after 24 h incubation (Fig. 8a). The cell viability values of the nCHA/PEO/CS-21 and nCHA/PEO/CS-11 scaffolds were considerably high in all scaffold concentrations (Table 5). Pursuing ISO 10993-5, the cell viability values of the nCHA/PEO/CS-21 and nCHA/PEO/CS-11 scaffolds perpetuated a cell viability value that exceeded the non-toxic density level of 80%,^67^ suggesting that the nCHA/PEO/CS-21 and nCHA/PEO/CS-11 scaffolds considerably had no cytotoxicity. The cell viability values of the nCHA/PEO/CS-11 scaffold were significantly higher than those of the nCHA/PEO/CS-21 (*p* < 0.001), suggesting that the higher content of chitosan within the nCHA/PEO/CS-11 scaffold enhanced the cytocompatibility since chitosan-based nanoparticles reportedly could increase the cell interaction by osteoconductive binding between positively charged surfaces of the amino group in the chitosan and negatively charged cell membrane.^68^ Hence, the nCHA/PEO/CS-11 scaffold induced better cell response for enhancing cell activity.

**Table 5:** Average cell viabilities of MC3T3E1 cells against the nCHA/PEO/CS-21 and nCHA/PEO/CS-11 scaffolds

No.	Scaffold concentration (μg mL^−1^)	Cell viability (%) (mean ± SD)		*p*-Value
		nCHA/PEO/CS-21	nCHA/PEO/CS-11	
1	7.81	89.5 ± 0.39	93.8 ± 0.28	0.001
2	15.6	88.8 ± 0.47	93.5 ± 0.17	
3	31.3	88.3 ± 0.49	93.1 ± 1.80	
4	62.5	87.9 ± 0.58	92.4 ± 1.27	
5	125	85.3 ± 0.41	90.8 ± 0.26	
6	250	84.4 ± 0.66	89.9 ± 0.58	
7	500	83.7 ± 0.29	89.8 ± 0.89	
8	1000	81.6 ± 1.33	85.9 ± 0.87	

For subsequence cytotoxicity analysis, the IC_50_ of the nCHA/PEO/CS-21 and nCHA/PEO/CS-11 scaffolds were examined statistically using non-linear curve fitting. The fitted non-linear curve on the average cell viability of the nCHA/PEO/CS-11 scaffold precisely outpaced those of the nCHA/PEO/CS-21 scaffold in all diluted scaffold concentrations (Fig. 8b). The IC_50_ values of the nCHA/PEO/CS-21 and nCHA/PEO/CS-11 scaffolds were estimated at 60.1 × 10^6^ and 20.8 × 10^6^ μg mL^−1^, respectively (Fig. 8c). Hence, the IC_50_ values of the nCHA/PEO/CS-21 and nCHA/PEO/CS-11 scaffolds were considerably high, suggesting that both scaffolds were highly safe to osteogenic cell for bone tissue repair. These results show that the highly osteoconductive nCHA in the scaffold could control the cytotoxic level in the high scaffold concentration.

**Fig. 9:**
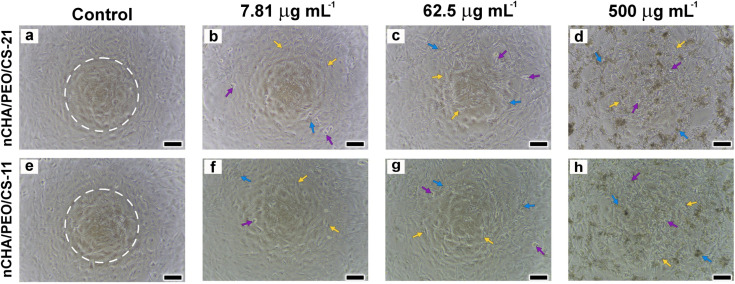
Morphology of MC3T3E1 cells after 24 incubation in conditioned medium with serial concentrations of (a–d) nCHA/PEO/CS-21 and (e–h) nCHA/PEO/CS-11 scaffolds. The white dashed circle, orange, purple, and blue arrowheads indicate cell cluster, life cells, dead cells, and scaffold particles, respectively. Scale bars: 100 μm (a–h).

The morphology of the well-connected MC3T3E1 cells formed a cell network in control and several diluted scaffold concentrations (Fig. 9a–h). Cell morphology in control formed several sub-confluent structures up to 80%, mostly clustered (Fig. 9a and e). As the scaffold concentration increased, most cells were still alive and highly viable with a fibroblastic structure, even though a few round-shaped cells appeared dead. The number of life cells in the nCHA/PEO/CS-11 scaffold (Fig. 9f–h) was seemingly higher than those in the nCHA/PEO/CS-21 scaffold (Fig. 9b–d), dealing with the cell viability values (Table 5). Hence, the nCHA/PEO/CS-11 was preferably suitable for use in bone regeneration. The higher content of biocompatible PEO/CS polymeric network within the nCHA/PEO/CS-11 scaffold triggered higher cell activity due to the presence of osteoconductive binding of amino group on the PEO/CS surface, thereby facilitating enhanced interaction with the cell membrane.^69,70^ Owing to its cytocompatibility, the nCHA/PEO/CS-11 scaffold is preferred in promoting bone regeneration.

#### 3.3.5. *In vitro* cell adhesion on the surfaces of nCHA/PEO/CS-21 and nCHA/PEO/CS-11 scaffolds

**Fig. 10:**
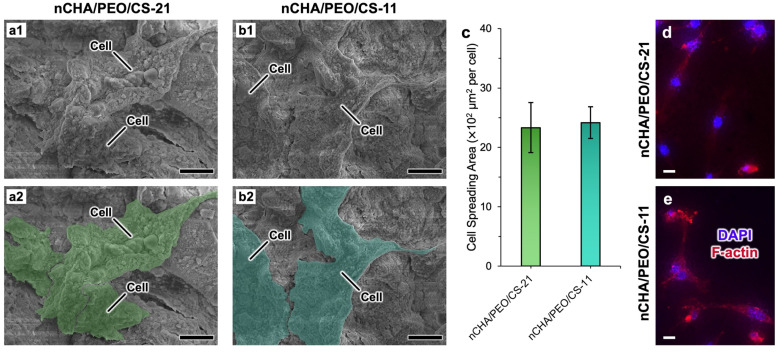
Morphology of adherent MC3T3-E1 cells on the surfaces of (a1) nCHA/PEO/CS-21 and (b1) nCHA/PEO/CS-11 scaffolds. (a2 and b2) Cells highlighted in the panels a1 and b1. (c) Cell spreading area on the surfaces of nCHA/PEO/CS-21 and nCHA/PEO/CS-11 scaffolds. Cytoskeleton of adherent cells on (d) nCHA/PEO/CS-21 and (e) nCHA/PEO/CS-11 scaffold surfaces. Scale bars: 20 μm (a1–b2,d,e).

After 48 h incubation, the MC3T3E1 cells adhered to the surfaces of nCHA/PEO/CS-21 and nCHA/PEO/CS-11 scaffolds (Fig. 10a1 and b2). The cells exhibited a multilateral spindle shape with extended pseudopodia (Fig. 10a1 and b2). During osteogenesis, osteoblast cells will extend their pseudopodia toward their mineralized side, gradually becoming dendritic.^71^ This finding suggests that nCHA/PEO/CS-21 and nCHA/PEO/CS-11 scaffolds may promote osteogenesis. Referring to the quantitative analysis of cell adhesion, the surfaces of the nCHA/PEO/CS-21 and nCHA/PEO/CS-11 demonstrated great spreading opportunities for the cells (Fig. 10c). The arrangement of the cell skeleton, mainly comprising actin filaments, confirmed the extension of pseudopodia of osteoblast cells (Fig. 10d and e), which is a crucial factor in influencing cell function and communication.^72^ The above results show that both scaffolds effectively facilitated the adherence of cells on their surfaces by providing appropriate rough sites for cell anchoring and interlocking with the scaffold structure. Regardless of the scaffold structure, these cell adhesion results agree well with the cell viability above, confirming that the osteoconductive nCHA and PEO/CS network improve the initial cell adhesion on the scaffold.^72,73^ Since initial cell adhesion is a critical factor for triggering cell proliferation and differentiation,^71^ the cytocompatible scaffolds have been improved in initial cell adhesion, which may favor bone regeneration.

#### 3.3.6. ALP activity of the nCHA/PEO/CS-21 and nCHA/PEO/CS-11 scaffolds

**Fig. 11:**
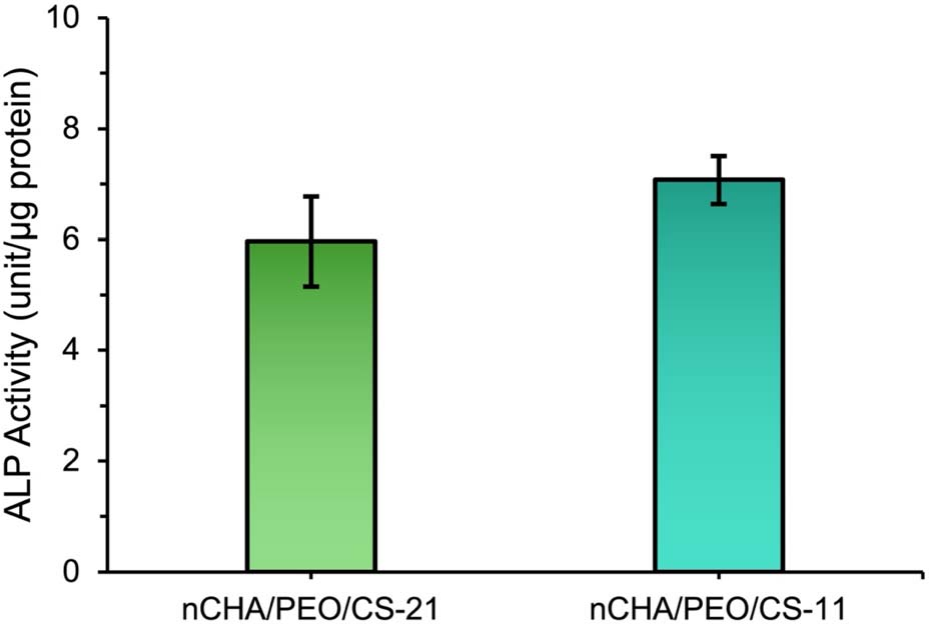
ALP activity of MC3T3-E1 cells cultured with the nCHA/PEO/CS-21 and nCHA/PEO/CS-11 scaffolds.

The quantitative evaluation for the ALP activity, a marker of early osteogenic differentiation, showed that the nCHA/PEO/CS-21 and nCHA/PEO/CS-11 scaffolds promoted osteogenic activity in MC3T3E1 cells, which was particularly stimulated by osteoinductive nCHA.^71^ Interestingly, the ALP activity of the nCHA/PEO/CS-11 was higher than that of the nCHA/PEO/CS-21 (Fig. 11), indicating that the modification with CS mainly enhanced osteoblast differentiation. These results are in accordance with similar reports that also demonstrated the performance of CS in promoting differentiation of osteoblast.^16,22^ The enhanced ability to induce osteogenic differentiation of cells proposes the nCHA/PEO/CS-11 to be highly preferable for use in facilitating bone regeneration.

## 4. Conclusions

This research achieved the fabrication of a porous nCHA/PEO/CS bone scaffold comprising a fibrous structure for mimicking native bone structure. The synthesized nCHA based on CFB demonstrated excellent physicochemical and morphological properties that are very close to apatite in natural bone. When nCHA was combined with PEO and CS in various concentrations by freeze-drying technique, the nCHA/PEO/CS scaffolds were obtained. The nCHA/PEO/CS scaffold had low crystallinity, which may encourage cell growth. The structure of all nCHA/PEO/CS scaffolds revealed that the nCHA nanoparticles were cross-linked within a fibrous-like structured PEO/CS network, which mimics the fibrous structure of extracellular matrix (ECM) in native bone. However, only the nCHA/PEO/CS-11 scaffold formed appropriate macro- and micropores with suitable macro- and micro-porosity that may enhance cell development, blood vessel formation, and nutrient perfusion. The nCHA/PEO/CS-11 scaffold also had sufficient compressive strength and well-regulated swelling behavior that may favor bone regeneration. The nCHA/PEO/CS-11 demonstrated high cytocompatibility and facilitated the adherence of MC3T3E1 cells onto the scaffold surface. The nCHA/PEO/CS-11 also promoted cell osteogenic differentiation. All in all, the developed nCHA/PEO/CS-11 scaffold was considerably promising in bone tissue engineering.

## Data availability

All data underlying the results are available as part of this article.

## Author contributions

M. R. Habiburrohman: conceptualization, methodology, investigation, data curation, writing – original draft, writing – review and editing. M. A. Jamilludin: conceptualization, data curation, writing – review and editing. N. Cahyati: conceptualization, data curation, writing – review and editing. N. Herdianto: funding acquisition, supervision. Y. Yusuf: validation, funding acquisition, supervision, project administration.

## Conflicts of interest

The authors declare that they have no known competing financial interests or personal relationships that could influence the work in this article.
